# Transferring genomics to the clinic: distinguishing Burkitt and diffuse large B cell lymphomas

**DOI:** 10.1186/s13073-015-0187-6

**Published:** 2015-07-01

**Authors:** Chulin Sha, Sharon Barrans, Matthew A. Care, David Cunningham, Reuben M. Tooze, Andrew Jack, David R. Westhead

**Affiliations:** School of Molecular and Cellular Biology, Garstang Building, University of Leeds, Leeds, LS2 9JT UK; Haematological, Malignancy Diagnostic Service, St James’s University Hospital, Leeds, UK; Section of Experimental Haematology, Leeds Institute of Cancer and Pathology, University of Leeds, Leeds, UK; Royal Marsden Hospital, Fulham Road, London, SW3 6JJ UK

## Abstract

**Background:**

Classifiers based on molecular criteria such as gene expression signatures have been developed to distinguish Burkitt lymphoma and diffuse large B cell lymphoma, which help to explore the intermediate cases where traditional diagnosis is difficult. Transfer of these research classifiers into a clinical setting is challenging because there are competing classifiers in the literature based on different methodology and gene sets with no clear best choice; classifiers based on one expression measurement platform may not transfer effectively to another; and, classifiers developed using fresh frozen samples may not work effectively with the commonly used and more convenient formalin fixed paraffin-embedded samples used in routine diagnosis.

**Methods:**

Here we thoroughly compared two published high profile classifiers developed on data from different Affymetrix array platforms and fresh-frozen tissue, examining their transferability and concordance. Based on this analysis, a new Burkitt and diffuse large B cell lymphoma classifier (BDC) was developed and employed on Illumina DASL data from our own paraffin-embedded samples, allowing comparison with the diagnosis made in a central haematopathology laboratory and evaluation of clinical relevance.

**Results:**

We show that both previous classifiers can be recapitulated using very much smaller gene sets than originally employed, and that the classification result is closely dependent on the Burkitt lymphoma criteria applied in the training set. The BDC classification on our data exhibits high agreement (~95 %) with the original diagnosis. A simple outcome comparison in the patients presenting intermediate features on conventional criteria suggests that the cases classified as Burkitt lymphoma by BDC have worse response to standard diffuse large B cell lymphoma treatment than those classified as diffuse large B cell lymphoma.

**Conclusions:**

In this study, we comprehensively investigate two previous Burkitt lymphoma molecular classifiers, and implement a new gene expression classifier, BDC, that works effectively on paraffin-embedded samples and provides useful information for treatment decisions. The classifier is available as a free software package under the GNU public licence within the R statistical software environment through the link http://www.bioinformatics.leeds.ac.uk/labpages/softwares/ or on github https://github.com/Sharlene/BDC.

**Electronic supplementary material:**

The online version of this article (doi:10.1186/s13073-015-0187-6) contains supplementary material, which is available to authorized users.

## Background

Gene expression patterns represent an attractive molecular phenotype for the classification of cancer [1–4]: they represent the functional state of the cancer cell that results from the perturbation of cellular processes such as signal transduction and genetic regulation, and whose underlying cause may be mutations or other changes in the cancer cell genome [4]. DNA microarrays have made gene expression measurements at the whole genome scale affordable for routine clinical diagnostics, and this has led to the development of gene expression signatures that may inform prognosis or treatment [5–8]. Blood cell cancers, leukaemia and lymphoma, are particularly attractive targets for gene expression signatures since they result from cells undergoing a complex pathway of differentiation, where cellular identity is largely defined by the pattern of gene expression, and where errors in differentiation or maturation are reproducibly manifest in cancers as aberrant patterns of gene expression [9]. Despite this, transfer of gene expression signatures into clinical practice has not proved straightforward [10, 11]. Different measurement technologies have emerged (e.g. microarrays, RT-PCR and RNA-seq) but, until recently, these have not been applicable to routine samples that are mainly formalin fixed and paraffin embedded (FFPE) in most centres. Furthermore, reproducibility between laboratories has proved challenging [12]. Equally, continual improvements in methodology, although welcome, raise the issue of transferability of signatures to newer platforms and can frustrate the clinical need for robust and fixed standards [13, 14]. Here we present a case study in the transfer of gene expression classifiers from the research literature into clinical practice.

We have adopted the example of Burkitt lymphoma (BL). This is a highly proliferative neoplasm that occurs sporadically in North America and European countries, but also has a variant associated with HIV infection and an endemic form common in Africa which is associated with Epstein–Barr virus (EBV) [15]. The criteria used to establish a diagnosis of BL have varied since its original description based on morphologic grounds in the endemic form, but it is now accepted that it is associated with translocation between the *MYC* oncogene and immunoglobulin gene [16], normally in the absence of chromosomal translocations involving oncogenes associated with diffuse large B cell lymphoma (DLBCL) [17, 18], and more recent studies have revealed further commonly associated mutations [19–21]. This is a case study of high clinical relevance, since treatment of BL requires intense chemotherapy [e.g. R-CODOX-M/IVAC; rituximab, cyclophosphamide, vincristine (known as Oncovin), doxorubicin methotrexate, ifosfamide, etoposide (known as Vepesid) and cytarabine ( known as Ara-C) [22], while in contrast DLBCL outcome is not improved by intensification of chemotherapy and is treated with a milder regime as first line therapy (e.g. R-CHOP; rituximab, cyclophosphamide, doxorubicin (known as hydroxydaunomycin), vincristine (known as Oncovin), prednisolone) [23]. However, a group of cases which are introduced as “B cell lymphoma, unclassifiable, with features intermediate between diffuse large B cell lymphoma and Burkitt lymphoma” [24] has received increased attention. These are likely to share some but not all pathogenetic features of classic BL, or arise as a result of alternative primary molecular events that nonetheless deregulate the common oncogenic pathways [25, 26]. This group appears to respond poorly to either intensive treatment or R-CHOP-like regimes [27–29], and the underlying mechanism remains largely unknown and the appropriate treatment still needs to be established.

Two seminal studies [30, 31] introduced gene expression-based classifiers to distinguish cases of BL and DLBCL based on data sets from different array platforms. Hummel and co-workers [31] adopted an approach whereby the set of classic BL samples was systematically extended on the basis of overall similarity in gene expression patterns to less clear cases. This semi-supervised approach using 58 genes effectively defined a new class called ‘molecular Burkitt lymphoma’. On the other hand, Dave and coworkers [30] based their supervised Bayesian method on independent expert pathology assignment of cases to the BL/DLBCL classes, and created a classifier based on 217 genes. The two classifiers are thus different in nature: they depend on relatively large gene sets with limited overlap and can be viewed as different gene expression-based definitions of BL.

Here, starting from the above work, we investigate optimal classification algorithms and gene lists to recapitulate the original classifiers, and by examining the transferability of the optimal classifiers between data sets we effectively compare the definitions of BL applied in each data set and classifier. Our own clinical data are based on RNA extraction from FFPE samples using the Illumina DASL (cDNA-mediated Annealing, Selection, extension and Ligation) technology, while the above classifiers were based on RNA extracted from fresh-frozen samples and different Affymetrix arrays. RNA in FFPE samples is more degraded, and although experimental protocols are improving, the data from this source remain significantly more noisy, and the change of measurement platform could have an equally significant effect. Nevertheless, FFPE data are likely to be the clinical reality for the foreseeable future, particularly in diagnostic laboratories responsible for large geographical areas with many hospitals. We investigate the production of a classifier based on a reduced gene set that can be effectively transferred between different gene expression measurement platforms in publicly available data sets and our own clinical data, and make a preliminary assessment of its likely clinical utility.

## Methods

### Data sets

The data sets used in this study are summarized in Table 1. Five public data sets were downloaded from the Gene Expression Omnibus [32]. GSE4732 was split into two subsets derived from different array platforms, here referred to as GSE4732_p1 and GSE4732_p2. Classifier development employed GSE4732_p1 and GSE4475, and the other data sets were used in testing transferability of classifiers.

**Table 1:** Data sets summary

GEO accession	Group	Samples	Probes	Platform
GSE4732_p1	Dave et al. [30]	54 BL, 249 DLBCL	2745	Custom Affymetrix Lympho-Chip
GSE4732_p2	Dave et al. [30]	33 BL, 66 DLBCL	54,675	Affymetrix HG-U133 plus2.0
GSE4475	Hummel et al. [31]	44 mBL, 48 intermediate, 129 non-mBL	22,283	Affymetrix HG-U133A
GSE10172	Klapper et al. [56]	13 mBL, 9 intermediate, 14 non-mBL	22,283	Affymetrix HG-U133A
GSE26673	Piccaluga et al. [57]	13 eBL, 2 HIV-BL	54,675	Affymetrix HG-U133 plus2.0
GSE17189	Deffenbacher et al. [58]	4 HIV-BL, 13 HIV-DLBCL	54,675	Affymetrix HG-U133 plus2.0
	HMDS	41 BL, 206 DLBCL clinically diagnosed	24,526	Illumina WG-DASL Version_3
	HMDS	70 BL, 169 DLBCL clinically diagnosed	29,377	Illumina WG-DASL Version_4

*eBL* endemic BL, *GEO* Gene Expression Omnibus, *HMDS* Haematological Malignancy Diagnostic Service, *HIV-BL* HIV-related Burkitt lymphoma *HIV-DLBCL* HIV-related diffuse large B cell lymphoma *mBL* molecular BL defined in corresponding paper

We also included 249 FFPE samples (GSE32918) from a previous study [33], together with 93 samples from the same platform Illumina DASL version 3 array and 250 samples from version 4 arrays in this study. Technical replicates were assessed both within each platform and between two platforms to examine reproducibility and consistency. The quality of each sample was checked before further analysis and the details are described in Additional file 1. The new samples analyzed have been submitted to the Gene Expression Omnibus with accession number GSE69053.

### Ethical approval

This study is covered by standard NRES (National Research Ethics Service) ethics approval for Haematological Malignancy Diagnostic Service (HMDS; St James Hospital, Leeds) local cases and treatment was not modified as a consequence of the study. The re-analyses of data from the LY10 and RCHOP14/21 clinical trials are separately covered by each trial’s ethical approval. This research is fully compatible with the Helsinki declaration.

### Data preparation

Preparation was done in R. All Affymetrix data sets except GSE4732_p1 were processed with the affy package [34] from raw data, and expression summarization was done with the rma algorithm [35] with quantile normalization. Gene identifiers were mapped with hgu133a.db [36] and hgu133plus2.db [37] packages. GSE4732_p1 was generated by an older custom array format and for this we used normalized expression data and gene identifiers provided by the authors. Pre-processing (including quality control) and expression summarization for the Illumina data sets was done with the lumi package [38] applying a vst transformation [39] and quantile normalization. Where multiple probes represented the same gene, the expression for the gene was summarized with the average value. All gene symbols were then checked with HGNChelper package [40] and updated to the latest approved symbol if necessary.

### Classifier performance assessment

Performance of classifiers was assessed using standard measures (overall error rate, overall accuracy, precision and recall within each class). Unless otherwise stated, performance was assessed by tenfold cross-validation when considering performance within a particular data set. We also assessed transferability of classifiers by training on one data set and testing on another. Further detail of these processes is provided in the "Results" section.

### Classification algorithms

We tested a total of ten algorithms, Bayes Net, Naïve Bayes, libSVM, SMO, Neural Network, Random Forest, Function Tree, LMT (logistic model tree), REP Tree and J48 pruned tree within GSE4732_p1 and GSE4472, respectively, using the Weka [41] machine learning tool. Our aim was not to compare methods, but rather to find a method able to recapitulate to an acceptable level of accuracy the classifications within these data sets. All algorithms were thus given default parameters (except to use 100 trees for the Random Forest), and parameters were then subsequently optimized just for the algorithm chosen for the remainder of the work. Initial investigations of different algorithms were carried out separately within each of GSE4732_p1 and GSE4475. Both of these data sets are associated with a classifier developed by the authors, and we used the gene lists from these classifiers as initial feature sets for algorithms above.

### Parameter optimization

We optimized parameters for one classification method, the support vector machine (SVM) implemented in libSVM [42]. Four common kernels are implemented in libSVM and we chose the most commonly used and recommended, the radial basis function (RBF). In this case parameter optimization involves the kernel parameter γ and the trade-off parameter *c*. We used the automatic script easy.py provided in the libSVM for a parameter grid search to select the model parameters: the search range of *c* value was 2^−5^ to 2^15^ with a step of 2^2^, the range of γ values was 2^3^ to 2^−15^ with a step of 2^−2^ and the cross-validation fold was 5 [43]. Note that parameter optimization was carried out by cross-validation within the training data, avoiding potential over-fitting that could result from using the complete data set.

### Probability calculation

In the case of the SVM classifier applied to our Illumina data set, the BL probability is a posterior class probability obtained from libSVM, employing an improved implementation of Platt’s posterior probability function for binary classification [44].

### Classifier gene set comparison

Subsequent development of classifiers involved a number of gene lists derived from those used in the authors’ classifiers for GSE4732_p1 and GSE4475 by consideration of issues such as availability of a gene expression measure for the gene on all platforms, robustness to over-fitting, and transferability to unknown data derived from different measurement platforms, as detailed in "Results" and "Discussion". In addition, we also tested the ten genes [45] used in a recent classifier that employs data from the NanoString [46] platform.

### Cross-platform normalization

Z-score, rank and two more sophisticated methods, XPN and DWD [47, 48] implemented in the CONOR package [49], were used to examine the effect of different cross-platform normalization methods. Z-score normalization operates for each gene independently, producing a normalized expression value in each sample as z = (x − m)/s, where x is the un-normalized expression value of the gene and m and s are the mean and standard deviation of x over all samples. For rank normalization, r = R/N − 0.5 is the normalized value, where R is the rank of the sample with respect to the N other samples on the basis of the expression of the gene concerned. Z-score and rank normalization have potential deficiencies, but also have the advantage of being applicable to data from methods such as RT-PCR and NanoString, which are designed to measure the expression of only relatively small gene sets.

### Software implementation

The developed classifier was implemented in the BDC package using the R package mechanism [50], and is available from the authors. The package provides a list of options for classifier gene set, cross-platform normalization method and data set to train the model along with reasonable default settings.

## Results

### Comparison of data sets and existing classifiers

The two existing classifiers were developed within GSE4732_p1 and GSE4475, respectively. Table 2 summarizes the gene sets used in these classifiers, the total numbers of genes measured on the corresponding platforms and the overlaps of these gene sets. The two classifiers use substantially different gene sets, with limited overlap, and in neither case are expression measurements of all classifier genes available on the other platform. It is impossible, therefore, to test a straightforward re-implementation of either classifier on the data sets that were not used in its development. Our aim, therefore, was to construct new classifiers and gene sets, based on those already existing, which adequately recapitulate the results of existing classifiers but are applicable to all data sets.

**Table 2:** Numbers of genes in data sets and used in existing classifiers

	GSE4732_p1	GSE4475	Overlap
HGNC approved genes on platform	2411	12495	1913
Genes used in authors’ classifier	217	58	21
Classifier genes located in data^a^	214	58	21
Classifier genes available in other data set^b^	172	28	-

^a^We were unable to locate all reported classifier genes in GSE4732_p1

^b^Dave et al. [30] classifier genes available in GSE4475 and Hummel classifier genes in GSE4732_p1

*HGNC*: HUGO Gene Nomenclature Committee, gene symbols from previous studies are checked and updated by HGNChelper package

### Recapitulation of existing classifications

**Fig. 1:**
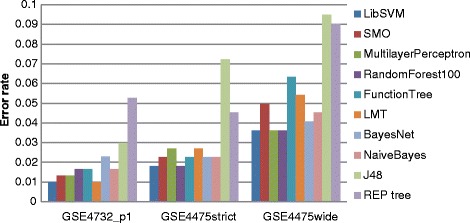
Performance of different machine learning algorithms with two previous data sets. Overall error rates (tenfold cross-validation within the data set GSE4732_p1, GSE4475_strict and GSE4475_wide, respectively) for the binary classification problem using a range of machine learning methods (LibSVM, SMO, MultilayerPerceptron, Random Forest, Function Tree, LMT, BayesNet, NaiveBayes, J48 and REP Tree, all implemented in Weka machine learning tool) with default parameters. In GSE4475 we consider two possible definitions of BL, strict (cases for which the authors give a BL probability of >0.95) and wide (BL probability >0.5). Classifiers are tested with the gene sets employed in the original papers for these data sets (214 genes for GSE4732_p1, 58 genes for GSE4475 strict and wide definition)

We developed classifiers using feature sets corresponding to the 214 gene list from the original classifier in GSE4732_p1, and the 58 gene list from the original classifier in GSE4475. Figure 1 shows the performance of a range of machine learning methods in both data sets (for detailed figures see Table S1 in Additional file 2). In GSE4732_p1 it is possible to achieve very low overall error rates of around 1 %. In GSE4475 we investigated two definitions of BL: BL probability assigned by the authors as >0.95 (strict) and >0.5 (wide), assigning other samples as DLBCL. Using the strict definition again very low error rates are possible (<2 %). On the other hand errors are larger with the wider definition, indicating that the classes are less well defined in terms of gene expression when this approach is adopted, and arguing in favour of using the stricter definition. Overall, given the level of uncertainty in the actual classification of intermediate cases, we consider that these results reproduce the previous work at a level sufficient to support further investigations. Based on relative performance, we chose to use SVMs as implemented in libSVM [42] as our classifier method.

### Optimization of SVM parameters and classifier gene list selection

Motivated by the fact that no platform has gene expression measurements for all the genes used in either original classifier, and aiming to reduce gene lists where possible because classifiers based on fewer features are less complex and less susceptible to over-fitting, we next sought to optimize the gene list for our classifier. At the same time we investigated the effect of optimizing SVM parameters. We considered further gene lists based on the existing classifiers: the 21 genes common to both original classifiers; the 28 genes for which measurements are available in GSE4732_p1 and are part of the classifier used in GSE4475; and the 172 genes that are part of the classifier genes used in GSE4732_p1 and available in GSE4475. A further list of 60 genes was newly identified by comparing the differentially expressed genes of the high confidence cases in each data set (which is 45 BL against 232 DLBCL in GSE4732_p1, and 44 mBL (molecular BL defined by the author)against 129 non-mBL in GSE4475; further details are given in Additional file 1).

**Fig. 2:**
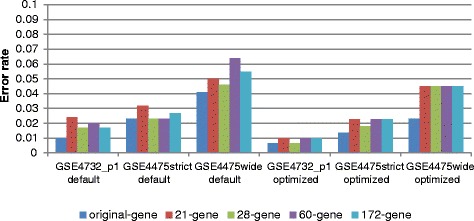
Performance of different gene sets built with libSVM algorithms. Overall error rates (tenfold cross-validation within the data sets GSE4732_p1, GSE4475strict and GSE4475wide, respectively) for binary classification problems using the gene sets described in the text: original refers to the gene sets used in Fig. 1; 21 genes are those used in both previous classifiers; the 28 genes for which measurements are available in GSE4732_p1 and are part of the classifier used in GSE4475; the 172 genes that are part of the classifier genes used in GSE4732_p1 and available in GSE4475; and 60 newly identified genes in this article. Classifiers were built with libSVM under default and optimized parameters, respectively

The results presented in Fig. 2 show that optimization of SVM parameters results in a modest (up to around 1 %) increase of accuracy over the use of default parameters. More importantly they show conclusively that classifiers based on small gene lists perform at least as well as their larger counterparts. The 28 gene list matches the performance of the full list in both data sets with only insignificant reductions in accuracy and was selected for future work. We also tested a recently published list of ten genes [45] developed with NanoString data. This list is insufficiently represented on the platform used in GSE4732_p1 with only six genes. We found it to perform similarly to our 21/28 gene lists in GSE4475 (Table S2 in Additional file 2), but in the absence of applicability to other test data sets we did not consider this gene list further and the five gene lists used to test the classifiers are provided in Additional file 3.

### Transfer of classifiers between data sets

Normalization of data to produce an expression measure that is comparable between platforms is an essential first step in producing transferable classifiers. We compared four cross-platform normalization methods, Z-score, Rank, XPN and DWD. The Z-score and Rank methods are the least sophisticated, but could be applied to data for small numbers of genes measured by most technologies. The other methods are more sophisticated and there is evidence that they perform better in some applications [32, 49], but they require measurements of many genes, such as those typically produced by microarrays. Table 3 shows the results of training a 28 gene SVM classifier on either GSE4732_p1 or GSE4475 and testing it on other data sets using different data normalization methods. All methods give similar results under the same training and test conditions, indicating that it is of no disadvantage to adopt one of the less sophisticated methods.

**Table 3:** Error rates for classifiers trained on one data set and tested on other public data sets

	BL error rate^a^				DLBCL error rate^a^			
Normalization	Z-score	Rank	XPN	DWD	Z-score	Rank	XPN	DWD
Train GSE4732_p1: test on other data sets below								
GSE4475 (strict)^b^	0.09	0.09	0.09	0.09	0.017	0.017	0.006	0
GSE4732_p2	0.182	0.212	0.152	0.152	0	0	0	0
GSE10172 (strict)^b^	0.231	0.308	0.385	0.308	0	0	0	0
GSE26673 eBL	0.615	0.692	0.846	0.384				
GSE26673 and GSE17189 HIV-related	0.833	1	1	0.667	0	0	0	0
Train GSE4475 strict BL definition: test on other data sets below								
GSE4732_p1	0.04	0.04	0.04	0.04	0.012	0.008	0.012	0.012
GSE4732_p2	0.303	0.333	0.273	0.273	0	0	0	0
GSE10172 (strict)	0.154	0.154	0.308	0.154	0	0	0	0
GSE26673 eBL	0.615	0.538	0.769	0.538				
GSE26673 and GSE17189 HIV-related	0.833	0.833	1	0.833	0	0	0	0
Train GSE4475 wide BL definition: test on other data sets below								
GSE4732_p1	0.02	0.02	0.02	0.02	0.04	0.05	0.06	0.07
GSE4732_p2	0.06	0.03	0.03	0.03	0.015	0.015	0.015	0.015
GSE10172 (strict)	0.078	0.078	0	0.078	0.043	0.043	0	0.043
GSE26673 eBL	0.154	0.154	0.308	0.154				
GSE26673 and GSE17189 HIV-related	0.5	0.333	0.833	0.5	0	0	0	0

^a^Error rate is (1 − Recall) value for the indicated class [Recall = True positives/(True positives + False negatives)]

^b^The sample in this data set is assigned to mBL, intermediate, non-mBL categories; here we set the strict BL definition as the standard which put intermediate and non-mBL together as the DLBCL class. *eBL* endemic BL, *mBL* molecular BL

First of all we considered the simple comparison of classifiers trained on one data set (GSE4732_p1 or GSE4475) and tested on the other. Table 3 shows that a classifier trained on GSE4732_p1 performs reasonably when tested on GSE4475 with the strict BL definition in the latter data set, giving error rates (recall) around 9 % for BL and <2 % for DLBCL. Conversely, training on GSE4475 (strict) and testing on GSE4732_p1 again gives good performance (errors around 4 % for BL and 1 % for DLBCL), indicating the classifier adopted on GSE4732_p1 corresponds to a BL criterion similar to the GSE4475 strict stratification. As would be expected, training with the wide definition of BL in GSE4475 reduces the BL error rate observed when testing on GSE4732_p1 to 2 % with a corresponding increase of the DLBCL error rate to around 5 %.

**Fig. 3:**
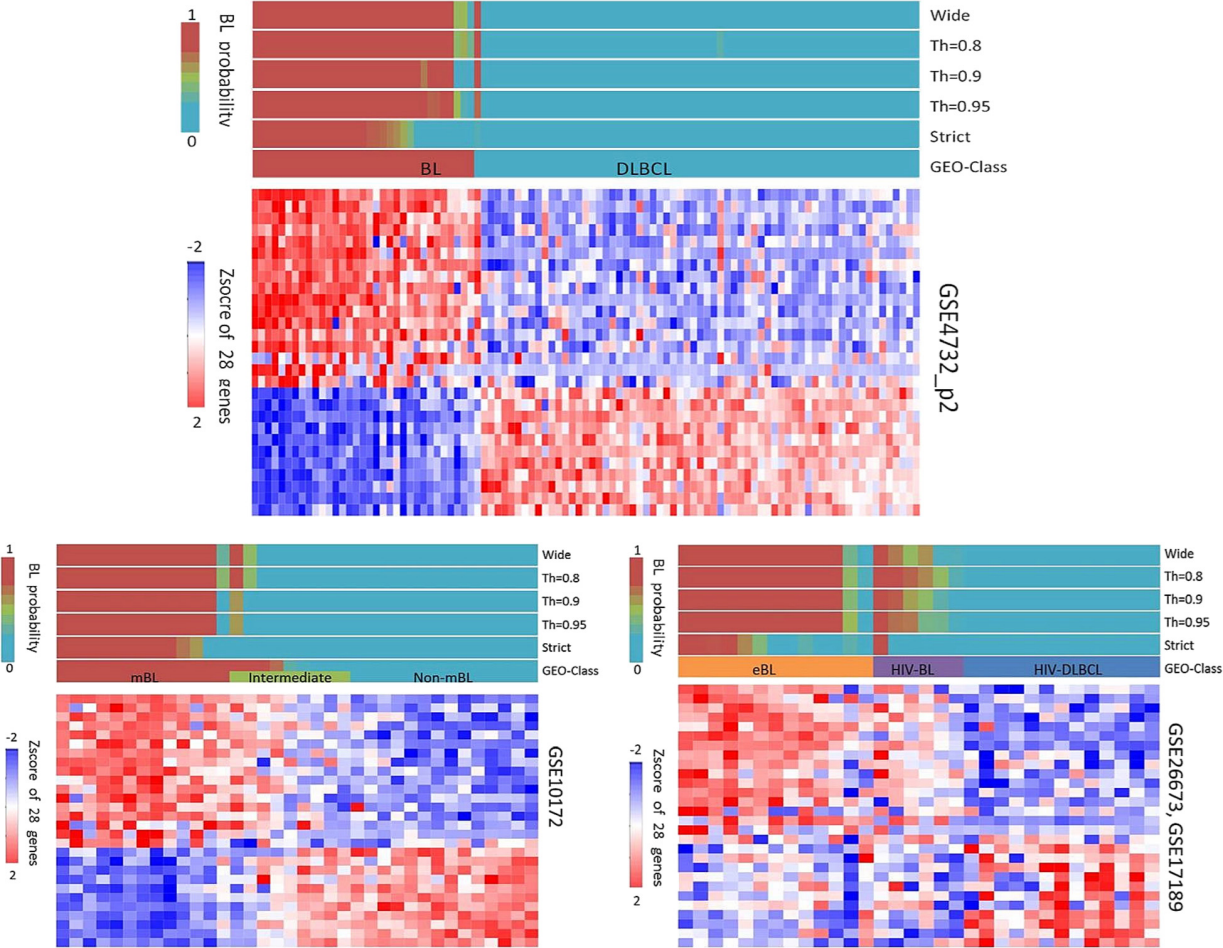
Performance of the classifier trained with different BL definitions with a heatmap of Z-score normalized 28 classifier gene expression values. Classification results of GSE4732_p2, GSE10172, GSE17189 and GSE26673 when the classifier was trained by a variety of thresholds, with a heatmap of the 28 classifier genes showing the Z-score normalized expression values. The training set threshold is adjusted according to data set GSE4475 and the class probability given to each sample by the original classifier; for example, training set *Th = 0.9* means only include the samples with a confidence over 0.9 in GSE4475 to train the classifier, and *Strict* and *Wide* refer to the strict and wide definition used previously. In test set GSE10172, the *GEO-Class* bar shows both the class label and BL probability from the original data set for each sample. The figure shows that when trained with the GSE4475 strict data set, the classifier has a strict definition of BL similar to with GSE4732_p1 but not very effective in recognizing BLs in GSE4732_p2 nor endemic BL (*eBL*) and HIV-related BL cases (*HIV-BL GEO* Gene Expression Omnibus

The performance of the above classifiers on other available data sets is also reported in Table 3. GSE4732_p2 is formed from a subset of the samples in GSE4732_p1 but with measurements from a different array platform (Table 1). It is surprising, therefore, that the classifier trained on GSE4732_p1 performs relatively poorly on this data set (BL error rates 15–21 % depending on normalization method), and the classifier trained on GSE4475 performs worse (BL error rates of 27–33 %). This effect is explored more thoroughly in Fig. 3 (top panel), which illustrates how different definitions of BL in the training data (GSE4475) affect the classifier. It is clear that with respect to this data set, the two consistent classifiers developed above adopt a narrower definition of BL, assigning cases with a weaker BL signal to the DLBCL category, and that a better classification result can be obtained by using a wider BL definition in the training set.

GSE10172 is a smaller data set generated by the group (Klapper, Molecular Mechanisms in Malignant Lymphomas Network Project of the Deutsche Krebshilfe) who produced GSE4475. Classifiers trained on either GSE4475 (strict) or GSE4732_p1 produce zero error rate for DLBCL cases but higher errors for BL: however, this is a relatively small data set and these findings may not be significant. Nevertheless, it is again the case that the classifier trained on the wide definition of BL in GSE4475 does produce a more accurate classification in GSE10172 (Fig. 3, bottom left panel), according to the classification given in that data set.

GSE17189 and GSE26673 are different in character, containing endemic BL (eBL) and HIV-related BL cases in contrast to the sporadic cases from the other data sets. Table 3 shows that the two classifiers trained with strict definitions of BL perform poorly with this data (BL error rate > 50 %). The lower right panel of Fig. 3 shows that cases of eBL have a similar gene expression pattern to the sporadic cases but generally with a weaker signal, explaining the high error rates from the strictly trained classifiers and the improvement in this when a wider definition is adopted. Many HIV-related BL cases on the other hand appear to have gene expression patterns related at least as strongly to DLBCL cases as they are to sporadic BLs and do not classify as BL with any choice of training data. Although sharing many pathologic features with sporadic BL, the eBL and HIV-related BL cases do have a distinct pathogenesis and gene expression. Some classifiers can recognize eBL seemingly well, but we suggest that training these classifiers on data for sporadic BL and applying it to eBL or HIV-related BL would not be advised. Given the distinct clinical settings of these disease variants, this does not pose a significant issue in relation to development of an applied gene expression-based classification tool.

To conclude, these studies show that despite using substantially different methods and genes, classifications within GSE4732_p1 or GSE4475 represent a largely consistent definition of BL that can be used as a basis for a classifier that uses fewer genes and transfers well between the two data sets. While this classifier does not apparently perform as well on other smaller and more diverse data sets, inconsistencies are largely related to intermediate cases and depend on where the boundary between classes is placed in a spectrum of cases in the training data. A similar test of the training set effect on GSE4475_p1 is shown in Additional file 4.

### Illumina DASL data sets

**Fig. 4:**
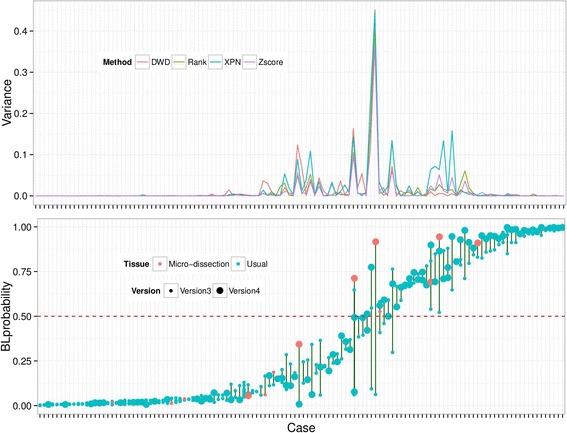
Classification consistency of the replicates from different platforms. *Top*: the variance of all replicate samples from the same patient when the data are normalized by Z score, Rank, DWD, and XPN methods, respectively. *Bottom*: the BL probability of each replicate (either has replicates in only one version or has replicates in each version) of the corresponding patient: bigger dots indicate version 4 data, smaller dots version 3 data, orange dots refer to micro-dissected tissue, and green dots are normal dissected tissue

Following the above investigations, we trained a 28 gene-based SVM, the BL and DLBCL classifier BDC, on the GSE4475 data set with a BL probability threshold of 0.95, and applied it to our Illumina data sets (Table 1) using several cross-platform normalization methods. Despite the results on the smaller data sets above indicating some advantage to a wider definition of BL, we preferred in this case the stricter definition (*p* = 0.95) because of its stronger consistency within and between the two larger data sets that were used in training studies. Of 592 samples in the version 3 and version 4 data together, 556 (93.9 %) have the same classification independent of normalization methods. For some cases the data sets contain replicates; 124 cases have a replicate on version 3 and version 4 together (including cases replicated within each version and some cases that are not replicated within a version but that have data from both versions). The variance of the BL probability of the total 124 replicates is given in Fig. 4 (top). Again this shows that if replicates show large variability, this is largely independent of normalization method. The Z-score normalization produces the smallest overall variance, and this was used subsequently.

The detailed results for all replicated cases are shown in Fig. 4 (bottom). This shows that the cases where the BL probability is most variable between replicates tend to be intermediate cases with BL probabilities closer to 0.5. It is also clear that version 4 data (with improved initial mRNA reverse transcription) generally give a stronger BL signal (BL probabilities closer to 1.0), probably reflecting better experimental treatment of BL samples, which, by their very nature, are more prone to significant degradation. Finally, it is clear that some of the larger variability between replicates occurs when one replicate is a tissue micro-dissection. Micro-dissection was performed on a subset of tumours following morphological inspection, with the aim of enriching for tumour content/and or the most adequately fixed area of the tissue. This would be expected to give stronger tumour-specific expression, as shown from previous experiments [33], and leads to a clearer classification of BL in the majority of cases.

### Comparison of original clinical diagnosis with gene expression-based classification

Our final BDC classification was based on reducing the Illumina data set to a single replicate for each case, choosing version 4 data in preference to version 3, micro-dissected tissue in preference to usual sampling, and otherwise choosing the newest array data. This gave a classification for 403 samples. The current clinical diagnosis of these samples is based on a range of immunophenotypic and molecular (fluorescent in situ hybridization, FISH) data as previously reported [28] and the agreement of this with the gene expression-based classification is shown in Table 4, where DLBCL diagnosed cases with a known chromosomal re-arrangement of the *MYC* gene are considered separately.

**Table 4:** Classification correlation with current clinical diagnosis

		Classified by BDC		
		BL	DLBCL	All
Diagnosed	BL	61 (85 %)	11 (15 %)	72
	DLBCL(*MYC*-rearranged)	13 (28 %)	34 (72 %)	47
	DLBCL	10 (4 %)	274 (96 %)	284

Generally there is a high level of agreement between the two diagnoses (85 % of clinically diagnosed BL cases classified as BL, and 96 % of clinically diagnosed DLBCL cases classified as DLBCL). Of the 11 clinical BL cases classified as DLBCL by BDC, three had classic BL characteristics, indistinguishable on conventional criteria from BL, but the remainder of the group included a high level of aberrant cases, with non-classic *MYC* rearrangement and/or discrepancies in immunophenotype. Of the ten diagnosed DLBCL cases predicted as BL, three showed a BL phenotype without *MYC* rearrangement. We also looked further at the small group diagnosed as DLBCL but with *MYC* rearrangement detected. This is a group of particular interest, many of which are now classified as “lymphoma with features intermediate between BL and DLBCL”, and though many studies have reported a poor prognosis, currently there is no specific treatment for this group [51–53]. In our data set (Table 5), 35 R-CHOP-treated cases in this group were classified into ten BL plus 25 DLBCL by BDC: the survival rate (remained alive or a complete remission from the treatment; for details see Table 5) of each class was 30 % and 68 %, respectively. Although these numbers are small, the survival difference observed suggests some advantage to gene expression classification that might eventually be examined in more detail in future trials. We note also that the survival rate (68 %) observed for intermediate cases classified as DLBCL by BDC is not significantly different from that for DLBCL as a whole (Kaplan-Meier, *p* = 0.4 compared with the R-CHOP-treated DLBCLs without *MYC* rearrangement. Full information is provided in the Gene Expression Omnibus data set).

**Table 5:** Detailed clinical information of 47 *MYC*-rearranged DLBCL cases

Sample ID^a^	BL prob^b^	Treatment^c^	Survival (years)/response^d^	BCL2, BCL6 rearrangement
13 cases with MYC-rearrangement classified as BL by BDC				
HMRN_52_v3_old	0.52	R-CHOP	Alive (4.46)	BCL2 rearranged
HMRN_30_v4_new	0.617	R-CHOP	Alive (1.9)	BCL2, BCL6 rearranged
RCH_125_v4_new	0.75	R-CHOP	Complete remission	BCL2, BCL6 rearranged
RCH_93_v4_new	0.665	R-CHOP	Persistent disease	BCL2 rearranged
HMRN_64_v4_old	0.716	R-CHOP	Died (2.14)	BCL2 rearranged
LY_43_v4_rep	0.662	R-CHOP	Died (0.45)	BCL2, BCL6 rearranged
HMRN_107_v4_old	0.745	R-CHOP	Died (0.63)	BCL2 rearranged
HMRN_34_v4_new	0.738	R-CHOP	Died (0.35)	BCL2 rearranged
HMRN_8_v4_old	0.561	R-CHOP	Died (0.95)	BCL2 rearranged
HMRN_74_v4_old	0.552	R-CHOP	Died (6.19)	BCL2 rearranged
LY_3_v4_new	0.777	CODOX-M/IVAC	Died (0.08)	BCL2 rearranged, BCL6 normal
HMRN_45_v4_rep	0.674	Unknown	Unknown	BCL2 rearranged
HMRN_56_v4_rep_2	0.711	Unknown	Unknown	BCL2, BCL6 rearranged
34 cases with MYC-rearrangement classified as DLBCL by BDC				
HMRN_49_v4_new	0.029	R-CHOP	Alive (1.08)	BCL6 rearranged
HMRN_47_v4_old	0.017	R-CHOP	Alive (6.40)	BCL6 rearranged
HMRN_112_v4_old	0.07	R-CHOP	Alive (6.31)	BCL6 rearranged
HMRN_52_v4_new	0.126	R-CHOP	Alive (1.22)	BCL2 amplified
HMRN_35_v4_new	0.168	R-CHOP	Alive (1.25)	BCL2 rearranged
HMRN_37_v4_new	0.008	R-CHOP	Alive (4.59)	BCL2 rearranged
HMRN_53_v4_old	0.032	R-CHOP	Alive (4.85)	BCL2 rearranged
HMRN_55_v4_new	0.136	R-CHOP	Alive (3.9)	BCL2 rearranged
HMRN_49_v4_old	0.01	R-CHOP	Alive (6.06)	BCL2 rearranged
HMRN_132_v3_old	0.023	R-CHOP	Alive (6.86)	BCL2, BCL6 normal
LY_35_v4_rep	0.016	R-CHOP	Alive (1.55)	BCL2 normal, BCL6 rearranged
LY_40_v4_rep	0.037	R-CHOP	Alive (1.59)	BCL2 normal, BCL6 rearranged
RCH_134_v4_new	0.086	R-CHOP	Complete remission	BCL6 rearranged
RCH_133_v4_new	0.148	R-CHOP	Complete remission	BCL2 rearranged
RCH_122_v4_new	0.19	R-CHOP	Complete remission	BCL2 rearranged
RCH_76_v4_new	0.162	R-CHOP	Complete remission	BCL2, BCL6 normal
RCH_120_v4_new	0.375	R-CHOP	Complete remission	BCL2, BCL6 normal
RCH_109_v4_new	0.008	R-CHOP	Persistent disease (died)	BCL6 rearranged
LY_37_v4_rep	0.071	R-CHOP	Died (0.95)	BCL2 rearranged, BCL6 normal
LY_17_v4_rep	0.15	R-CHOP	Died (1.04)	BCL2, BCL6 normal
HMRN_46_v4_rep	0.245	R-CHOP	Died (0.49)	BCL2 rearranged
HMRN_104_v3_old	0.009	R-CHOP	Died (0.39)	BCL2, BCL6 normal
HMRN_76_v4_old	0.195	R-CHOP	Died (0.66)	BCL2, BCL6 normal
HMRN_42_v4_new	0.088	R-CHOP	Died (1.81)	BCL2, BCL6 normal
HMRN_129_v4_old	0.02	R-CHOP	Died (0.37)	BCL2, BCL6 normal
LY_21_v4_rep	0.5	CODOX-M/IVAC	Died (0.14)	BCL2 rearranged, BCL6 normal
HMRN_172_v4_old	0.034	No active treatment	Died (0.02)	BCL2 rearranged
HMRN_48_v4_rep	0.015	No active treatment	Died (1.8)	BCL2 rearranged
HMRN_5_v3_old	0.082	No active treatment	Died (0.18)	BCL6 rearranged, BCL2 amplified
HMRN_23_v4_new	0.015	No active treatment	Died (0.09)	BCL2, BCL6 normal
HMRN_44_v4_rep	0.313	Died before treatment	Died (0.04)	BCL2 rearranged
HMRN_54_v4_rep	0.216	Died before treatment	Died (0.03)	BCL2 rearranged
HMRN_70_v4_rep	0.111	R-CHOP	Unknown	BCL6 rearranged
HMRN_18_v4_new	0.093	Unknown	Unknown	BCL2, BCL6 rearranged

^a^Samples are collected from two clinical trails as well as local cases in the Haematological Malignancy Diagnostic Service (HMDS; St James Hospital, Leeds). Sample IDs starting with RCH are records from R-CHOP-treated trials and those starting with LY [28] are records from a dose-modified CODOX-M/IVAC trial. The rest of the samples starting with HMRN are local patients from the HMDS database. Samples labelled with the _old suffix are cases submitted to the Gene Expression Omnibus (GEO) in previous studies, and those labeled with the _new suffix are cases not in the GEO database yet

^b^BL prob is the Burkitt lymphoma probability generated by BDC (Burkitt lymphoma and diffuse large B cell lymphoma classifier developed in our work)

^c^R-CHOP is usually used to treat DLBCL; CODOX-M/IVAC is a treatment usually for BL or BL-like patients

^d^In R-CHOP trials, all patients are evaluated with treatment response and this is used in the study

## Discussion

The work presented here provides an important step in establishing an optimized, parsimonious and open access gene expression-based classifier for BL. By using the results of one classifier and its associated data set for training, and the other as test data, we have shown that two substantially different classifiers in the research literature have a high degree of concordance and that their results can be recapitulated, at least within the level of uncertainty associated with intermediate cases. We have also shown that this unified classifier can be successfully applied to other public data sets and to data from routine clinical samples. In the context of our own clinical data, the classifier shows a high degree of concordance with the original diagnosis.

At a technical level, the reduction of the gene set compared with the original classifiers is a substantial advantage, making the classifier simpler and opening the possibility of using other measurement technologies such as quantitative PCR or NanoString in clinical applications. In addition, our detailed exploration of different training sets is noteworthy, since classifiers developed so far have largely been trained and tested within single data sets. Clearly the output of a classifier for borderline cases is critically dependent on the labelling of similar cases in the training data: our study maps the effect of changing training classification criteria in detail, and highlights differences in the classification of borderline cases between different data sets when examined in the context of gene expression criteria. Our final decision was to train the classifier on a two-way definition of BL based on the original class of GSE4475, but this nevertheless assigns fewer cases as BL than indicated in some other public data sets.

Other recent work in the field has also highlighted the possibility of using reduced gene sets [45, 54] for classification and also paraffin embedded samples, in these cases using data from the NanoString platform, which measures expression of a user-defined gene panel. It is an open question whether clinical use is better served by genome scale measurements (e.g. Affymetrix or Illumina arrays, RNA-seq) for each case, or possibly more precise measurements of just those genes needed for classification. However, the work reported here relies on genome scale measurements provided in publicly available data sets: this enabled our detailed comparison of different classifiers and their transferability, and the production of a consensus. This is not possible in general with NanoString data sets, since they seldom contain all the genes required by other classifiers. Our approach has been to leverage as much value as possible from existing data sets and previous classification work. We would support genome scale data generation from clinical samples in the future because it is of much greater utility in research and in the detailed comparison of competing methodologies.

Dependence on training data highlights the underlying difficulty in this and many similar studies, which is the lack of a ‘gold standard’ against which to evaluate new classifiers. Even though disease categories like BL and DLBCL have developed over many years with a variety of phenotypic and molecular diagnostic criteria, there are still a significant number of cases which are complex and neither expert pathological assessors nor recent molecular classifiers can effectively distinguish them. An alternative evaluation is to examine survival separation or treatment response, which is the primary clinical concern, and we used our own clinical data to examine outcome on the same treatment for cases where gene expression classification disagreed with the original diagnosis. Such discordant cases are relatively few even in a large data set, and the next step will be to make this evaluation in more cases as they become available. However, it is important to note that the treatment options in the setting of B-cell malignancies are likely to evolve at a high rate in the near future, and thus the use of clinical outcome with currently conventional therapy is likely to be an unstable parameter against which to assess the value of classification.

Our decision to develop a binary classifier for BL versus DLBCL, instead of introducing a third intermediate class, is related to the issues described above. Since there are only two main treatment regimes, a third class is not clinically useful. We prefer a classifier that makes a decision one way or the other on intermediate cases, bearing in mind that uncertainty is reflected in the associated class probabilities. It would be naïve to suggest that such a classifier could be the sole basis for treatment decisions, but it can effectively add to the weight of evidence a clinician might consider.

More recent findings have indicated new genetic distinctions between BL and DLBCL [20, 21, 55]. It remains an open question whether the diseases are better distinguished by these or a gene expression phenotype. However, it seems likely that a combination of both information sources as the basis of future classifiers could lead to increased robustness in the context of heterogeneous diseases and the inevitable noise associated with all measurements on clinical samples.

We have previously developed an applied gene expression-based classifier for the separation of DLBCL cases into so-called “cell of origin” classes in samples derived from FFPE material [33]. This tool is currently being applied in a routine clinical setting in the context of a phase 3 clinical trial, and the BDC tool developed in this work could be applied with this to provide a more complete diagnostic pathway in routine clinical practice.

## Conclusions

The identification of cases of BL is clinically critical. Classic cases of this disease are treated effectively with intense regimens but not with the standard treatment for DLBCL. However, an intense regimen is more costly, less convenient and unsuitable for weaker patients who may not withstand the toxic challenge. Intermediate cases therefore represent a significant difficulty. Our data show that it would be naïve to suggest that gene expression-based classification can solve this problem, but that it does have a potential role to play. We suggest that in cases with a standard diagnosis of DLBCL, gene expression could be used alongside other evidence and phenotypic features in deciding whether to treat with more intensive therapy. Future work should evaluate this suggestion, alongside the incorporation of genetic data in classification.

## Additional files

Additional file 1:
**Additional methods on gene selection and quality checking.**
Additional file 2:
**Additional tables of tested classifier results.**
Additional file 3:
**Gene sets tested in different classifiers.**
Additional file 4:
**Performance of the classifier trained with different BL definitions tested on GSE4732_p1 with a heatmap of Z-score normalized 28 classifier gene-expression values.** The training set threshold is adjusted according to data set GSE4475 and the class probability given to each sample by the original classifier; for example, training set *Th=0.9* means only include the samples that have a confidence over 0.9 in GSE4475 to train the classifier, and *Strict* and *Wide* refer to the strict and wide definition used previously. The GSE4475 (strict) trained classifier classifies cases similar to the original category in the paper, while other training sets would classify a small group of DLBCL cases as BL. However, the heatmaps of those cases exhibit similar expression patterns as classic BL, suggesting these are intermediate cases with less confidence for which class they belong to.

## Abbreviations

BDC: Burkitt lymphoma and diffuse large B cell lymphoma classifier
BL: Burkitt lymphoma
CODOX-M/IVAC: cyclophosphamide, vincristine (known as Oncovin), doxorubicin methotrexate, ifosfamide, etoposide (known as Vepesid) and cytarabine (known as Ara-C)
DASL: cDNA-mediated Annealing, Selection, extension and Ligation
DLBCL: diffuse large B cell lymphoma
eBL: endemic Burkitt lymphoma
FFPE: formalin fixed and paraffin embedded
HMDS: Haematological Malignancy Diagnostic Service
R-CHOP: rituximab, cyclophosphamide, doxorubicin (known as hydroxydaunomycin), vincristine (known as Oncovin), prednisolone
SVM: support vector machine
