# Supplementary material for: Transferring genomics to the clinic: distinguishing Burkitt and diffuse large B cell lymphomas
Source: Genome Med. 2015 Jul 1;7(1):64. doi: 10.1186/s13073-015-0187-6 (PMC4512160; doi:10.1186/s13073-015-0187-6)
Supplement: Additional file 1: — Additional methods on gene selection and quality checking. [file 13073_2015_187_MOESM1_ESM.docx]

# Additional Methods

### Self-identified genes

Apart from 4 gene sets derived from previous classifiers, we also identified a new gene set based on both GSE4732_p1 and GSE4475. And we only considered the high confidence samples in the previous classifiers to identify differentially expressed genes between BL and DLBCL. In the Dave classifier (GSE4732_p1), we compared against the consistent 44 BL versus 235 DLBCL agreed by both pathological and molecular diagnosis; and in the Hummel classifier (GSE4475) we only compared the 44 mBLs against the 129 non-mBLs. First 100 most significantly differentially expressed genes between the two groups in each data set were selected by limma package, and there are 54 genes overlap. The new identified gene set was consist of the 54 overlap genes with 6 genes that applied in both two previous classifiers but not picked in above selecting method.

### Quality Control of Clinical FFPE samples

Quality check involved the detected signal over all probes, housekeeping control probes, annealing control probes, cyc_hyb control probes, low stringency control probes and negative control probes. Arrays falling beyond 1.645 standard deviations from the mean of a criterion were considered failures at that criterion, and arrays were removed from the data set if they failed at probe detection or housekeeping genes or either two of annealing control probes, cyc_hyb control probes, low stringency control probes or negative control probes. And there are 97 and 119 samples removed from downstream analysis due to poor quality in Version 3 and Version 4 dataset respectively.
