# Supplementary material for: Transferring genomics to the clinic: distinguishing Burkitt and diffuse large B cell lymphomas
Source: Genome Med. 2015 Jul 1;7(1):64. doi: 10.1186/s13073-015-0187-6 (PMC4512160; doi:10.1186/s13073-015-0187-6)
Supplement: Additional file 2: — Additional tables of tested classifier results. [file 13073_2015_187_MOESM2_ESM.docx]

# Supplementary Tables

| **Supplementary Table 1: Accuracy of 10-fold cross-validation in two data sets** | | | | | | |
| --- | --- | --- | --- | --- | --- | --- |
|  | **GSE4732(1): Multi-Class** | **GSE4475: Multi-Class** | **GSE4732(1): Binary-Class** | **GSE4475: Strict** | **GSE4475: Wide** | **Average** |
| LibSVM | 88.45 | 91.86 | 99.01 | 98.19 | 96.39 | 94.78 |
| SMO | 88.45 | 92.77 | 98.68 | 97.74 | 95.03 | 94.534 |
| MultilayerPerceptron | 89.11 | 89.6 | 98.68 | 97.29 | 96.39 | 94.214 |
| RandomForest100 | 83.83 | 93.22 | 98.35 | 98.19 | 96.39 | 93.996 |
| FT | 86.47 | 88.24 | 98.35 | 97.74 | 93.67 | 92.894 |
| LMT | 82.51 | 86.88 | 99.01 | 97.29 | 94.57 | 92.052 |
| BayesNet | 77.23 | 89.6 | 97.7 | 97.74 | 95.93 | 91.64 |
| NaiveBayes | 77.56 | 88.24 | 98.35 | 97.74 | 95.48 | 91.474 |
| J48 | 73.27 | 81.91 | 97.03 | 92.77 | 90.5 | 87.096 |
| REP tree | 72.3 | 81.91 | 94.72 | 95.48 | 90.96 | 87.074 |

| ^1^GSE4732(1) Multi-Class includes 54 BL, 91 ABC (Activated B-Cell-like), 95 GCB (Germinal Centre B-cell-like), 33 PMBL (Primary Mediastinal B-cell Lymphoma), 30 Unclassified DLBCL, ; GSE4732(1) Binary-Class are 54 BL and 249 DLBCLs.  ^2^GSE4475 Multi-Class are 44 mBL, 48 intermediate and 129 non-mBL cases; GSE4475 strict are 44 BL versus 177 others; GSE4475 wide are 59 BL and 162 DLBCL divided by author assigned BL probability equals 0.5 |
| --- |

| **Supplementary Table 2: Overall accuracy of tested gene lists in building the classifier** | | | | | | | |
| --- | --- | --- | --- | --- | --- | --- | --- |
|  | **GSE4732 default** | **GSE4475strict default** | **GSE4475wide default** | **GSE4732 optimized** | **GSE4475strict optimized** | **GSE4475wide optimized** | **Average** |
| original-gene | 0.99 | 0.977 | 0.959 | 0.993 | 0.986 | 0.977 | 0.980 |
| 10-gene | 0.974 | 0.968 | 0.954 | 0.97 | 0.968 | 0.955 | 0.965 |
| 21-gene | 0.977 | 0.968 | 0.95 | 0.99 | 0.977 | 0.955 | 0.969 |
| 28-gene | 0.984 | 0.977 | 0.954 | 0.993 | 0.982 | 0.955 | 0.974 |
| 60-gene | 0.98 | 0.977 | 0.936 | 0.99 | 0.977 | 0.955 | 0.969 |
| 172-gene | 0.984 | 0.973 | 0.945 | 0.99 | 0.977 | 0.955 | 0.971 |
| *only 6 of the 10 NanoGene are found and tested in GSE4732(1) dataset | | | | | | | |
