# Supplementary figures and images for: Transferring genomics to the clinic: distinguishing Burkitt and diffuse large B cell lymphomas
Source: Genome Med. 2015 Jul 1;7(1):64. doi: 10.1186/s13073-015-0187-6 (PMC4512160; doi:10.1186/s13073-015-0187-6)

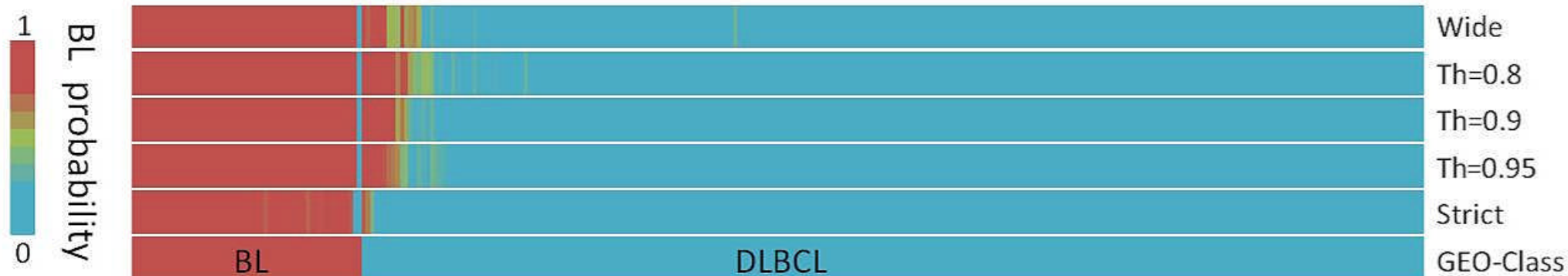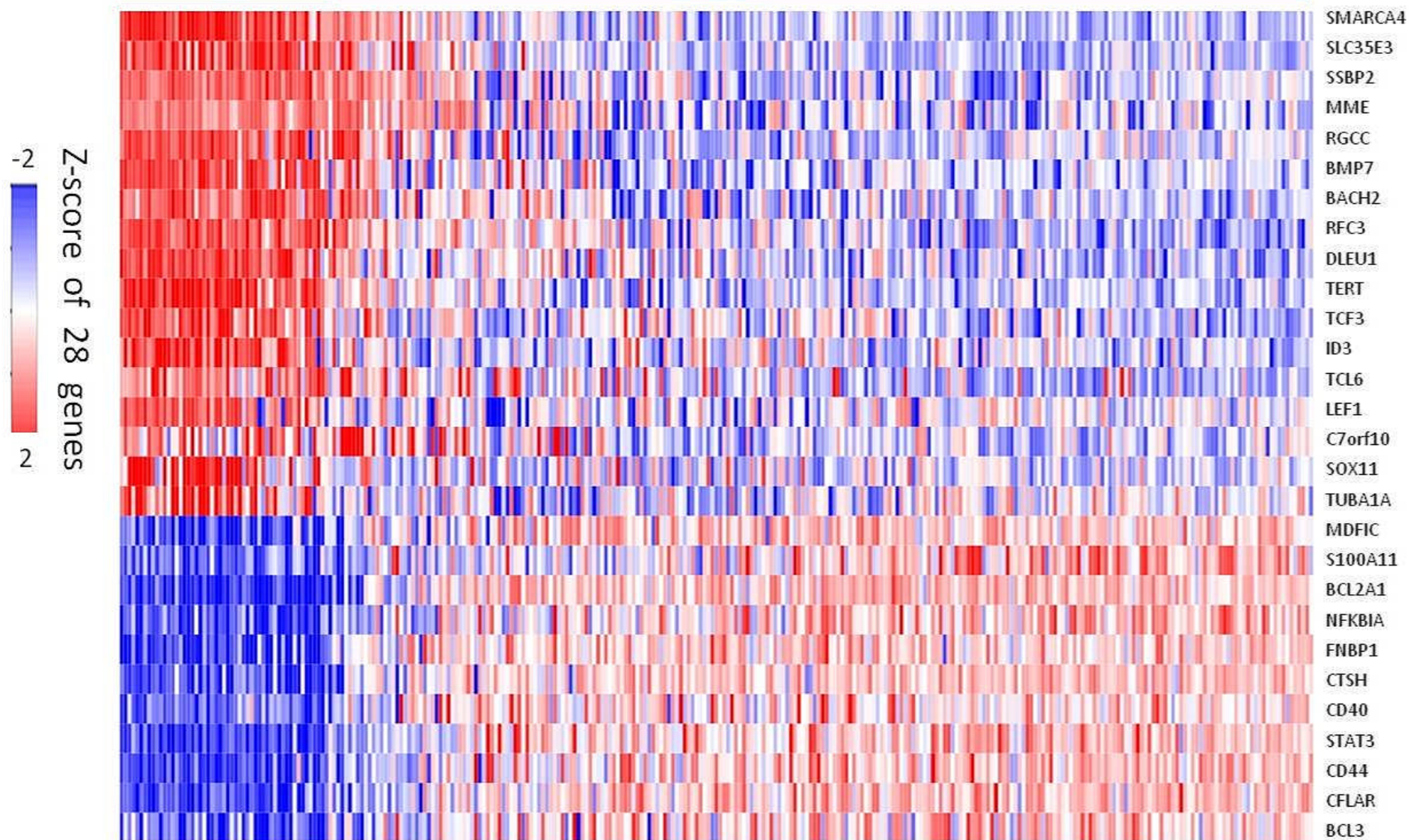

Supplement: Additional file 4: — Performance of the classifier trained with different BL definitions tested on GSE4732_p1 with a heatmap of Z-score normalized 28 classifier gene-expression values. The training set threshold is adjusted according to data set GSE4475 and the class probability given to each sample by the original classifier; for example, training set Th=0.9 means only include the samples that have a confidence over 0.9 in GSE4475 to train the classifier, and Strict and Wide refer to the strict and wide definition used previously. The GSE4475 (strict) trained classifier classifies cases similar to the original category in the paper, while other training sets would classify a small group of DLBCL cases as BL. However, the heatmaps of those cases exhibit similar expression patterns as classic BL, suggesting these are intermediate cases with less confidence for which class they belong to. [file 13073_2015_187_MOESM4_ESM.pdf]
